# Integrating science and the arts to deglobalise climate change adaptation

**DOI:** 10.1038/s41467-024-47400-7

**Published:** 2024-04-06

**Authors:** Marta Olazabal, Maria Loroño-Leturiondo, Ana Terra Amorim-Maia, William Lewis, Josune Urrutia

**Affiliations:** 1 Basque Centre for Climate Change, BC3, Bilbao, Spain; 2 Ikerbasque Science Foundation, Bilbao, Spain; 3 Independent artist and researcher, Bilbao, Spain

**Keywords:** Climate-change adaptation, Climate-change policy, Psychology and behaviour, Geography

## Abstract

Setting goals that are context-specific, relevant, and collectively shared is critical in adaptation. As necessary elements in target setting, imaginaries for adaptation and the language connected to them remain vague. Visuals produced through art-science collaborations can be great allies to (de)construct imaginaries and deglobalise discourses of adaptation.

## Adaptation that counts in local contexts

Adaptation to climate change is an ambiguous concept and, for this reason, a highly contested one. The use of unclear and general language in adaptation science and politics is behind this problem. For example, the language used by the Intergovernmental Panel on Climate Change (IPCC)^1^ to define adaptation is too technical and hazard-focused and it does not engage with local needs. Adaptation requires processes that act on context-specific vulnerabilities and risks^2^ and an awareness of the diversity of outcomes and impacts. An inflexible global framing of adaptation detached from context is often the reason why maladaptation, actions that inadvertently lead to negative outcomes from adaptation, emerges^3,4^. Deglobalising adaptation involves making it relevant to local contexts and bringing the discourse and practice back to the local sphere. This includes setting locally defined and relevant targets and goals, and developing local socio-political imaginaries of what good adaptation looks like.

As a result of the universally adopted technocratic discourse of adaptation, scientists, policymakers, and practitioners have experienced ongoing challenges in (1) producing a common understanding of what successful adaptation is^5^; (2) facilitating community-based and locally-led processes to adapt^6,7^; and (3) establishing concrete adaptation goals, particularly at the local level^8–10^. For example, goals related to ‘equity’ or ‘justice’ will remain useless unless we adequately factor in context-specific vulnerabilities, risks, perceptions, resources, politics, and history^11^. The same applies to other framings of effective adaptation such as sustainability, resilience, transformation, well-being, or maladaptation^12^. In addition to this, top-down framings for adaptation planning and quantitative data-driven adaptation can very easily overlook structural vulnerabilities at the local level and drive universal models of adaptation planning and decision-making that are not meant to work across every socio-political context. Imaginaries of good adaptation are not universal, and efforts in this direction are likely to fail. So, what does it mean and how to explain what it is to adapt? How to establish goals that have meaning in local contexts? And, how to move from policy ambiguity to meaningful transformative action?

## Visuals to deglobalise adaptation meanings

Language has so far been the key resource for awareness-raising, communication, planning, negotiation, and decision-making in the socio-political arenas of adaptation. In theory, language should not be limited to describing the present but also to imagining adaptation realities and challenging them to create disruptive pathways for action. At a time when adaptation had a limited role in policy discourses, language has been very useful in creating symbolism^13^ through universal abstractions regarding what adaptation involves (resilience, transformation, justice) or what it is not meant to involve (risk, maladaptation or vulnerability). However, it has not been successful in shaping imaginaries of what adaptation might look like on the ground. Two significant challenges hinder the use of language as an entry point for context-specific adaptation management: (1) its abstraction and technocratisation, and (2) its lack of local meaning. We here argue that, while the current language used in adaptation is a critical resource across stages of policy, planning, awareness and education, it alone cannot generate ownership and produce relevant action at the local level. Visuals are also necessary tools.

Art-based approaches, have an immense potential to help picture and communicate the future of nature–social interactions^14^. The future is complex and abstract, and literature on visual communication suggests that images have the power to make us engage at an affective level (involving the experiential processing system), which is fundamental in processing risk and uncertainty^15^. Furthermore, images can trigger action and can be useful to engage in an exercise to establish or disrupt ways of doing and responding^16^. Visuals can help engage and empower people who would normally feel excluded from traditional participatory processes, building more appropriate environments to enable just outcomes from adaptation. Here we argue that visuals, especially those co-created with local actors, can surpass language benefits in deglobalising the now too universal and abstract adaptation discourse. However, most non-language climate adaptation materials are typically in the form of graphics or maps, often produced through highly top-down data visualisation practices. At present, visuals for adaptation in grey literature, government policy and planning documents, and websites, are dominated by depictions of climate impacts rather than illustrated solutions or, more importantly, explanations of adaptation processes. We argue that the lack of adaptation visuals of context-specific climate-adapted futures is a significant barrier to gaining more conscious and thoughtful use of language. Further efforts need to be developed to make adaptation visuals more comprehensive and tangible across diverse contexts.

In this sense, art–science and art–policy collaborations are proliferating. However, in most cases, scientists, policymakers or adaptation practitioners seek out visual support only to help them engage the public with their work as an end-of-pipe approach^17^. The art-based approach that we suggest here, and that we feel necessary to deglobalise adaptation, emerges from a transdisciplinary approach and starts much earlier on in the adaptation management cycle, making the arts central to planning, decision-making, and communication.

## Lessons from IMAGINE Adaptation

The goal of the “Urban Imaginaries of Adaptation” study—developed as part of the European Research Council project IMAGINE Adaptation—is to gather local constructs of adaptation to enable a discussion on the tangible influence of technocracy on current adaptation practice and the imaginaries of a city adapting to climate change. The project navigated the challenges of using both language and visuals through the process of constructing these imaginaries. We first identified concepts related to adaptation in urban areas through literature and an expert survey, and we also gathered hundreds of images related to urban adaptation across media and grey literature. Using this groundwork as a baseline for multiple adaptation framings, we started an art–science collaboration between us, the researchers behind IMAGINE Adaptation, and an artist experienced in illustrative design to translate identified concepts into images. The ultimate goal was to translate abstract concepts (migration, loss and damage, nature-based solutions, legitimacy, intersectionality, data-driven governance, transformation, etc.) that are generally connected to adaptation into visuals that could communicate across many different contexts, thus helping to deglobalise the concept of adaptation. Around 100 urban adaptation practitioners and experts (local governments, non-governmental organisations, educational organisations, civic associations, and businesses) across the globe (from Mexico to Afghanistan, Greece to Sierra Leone) were invited to imagine their cities adapted to climate change through a series of 31 illustrations, created by the professional illustrator. The study covered cities and localities across world regions with different population sizes, development levels, vulnerabilities, climate risks and adaptation experiences. Participation involved looking at these images and deciding which most (and least) represent participants’ views of adaptation in their city.

However, working with images does not come without its challenges.

### Multiple and diverse audiences

**Fig. 1:**
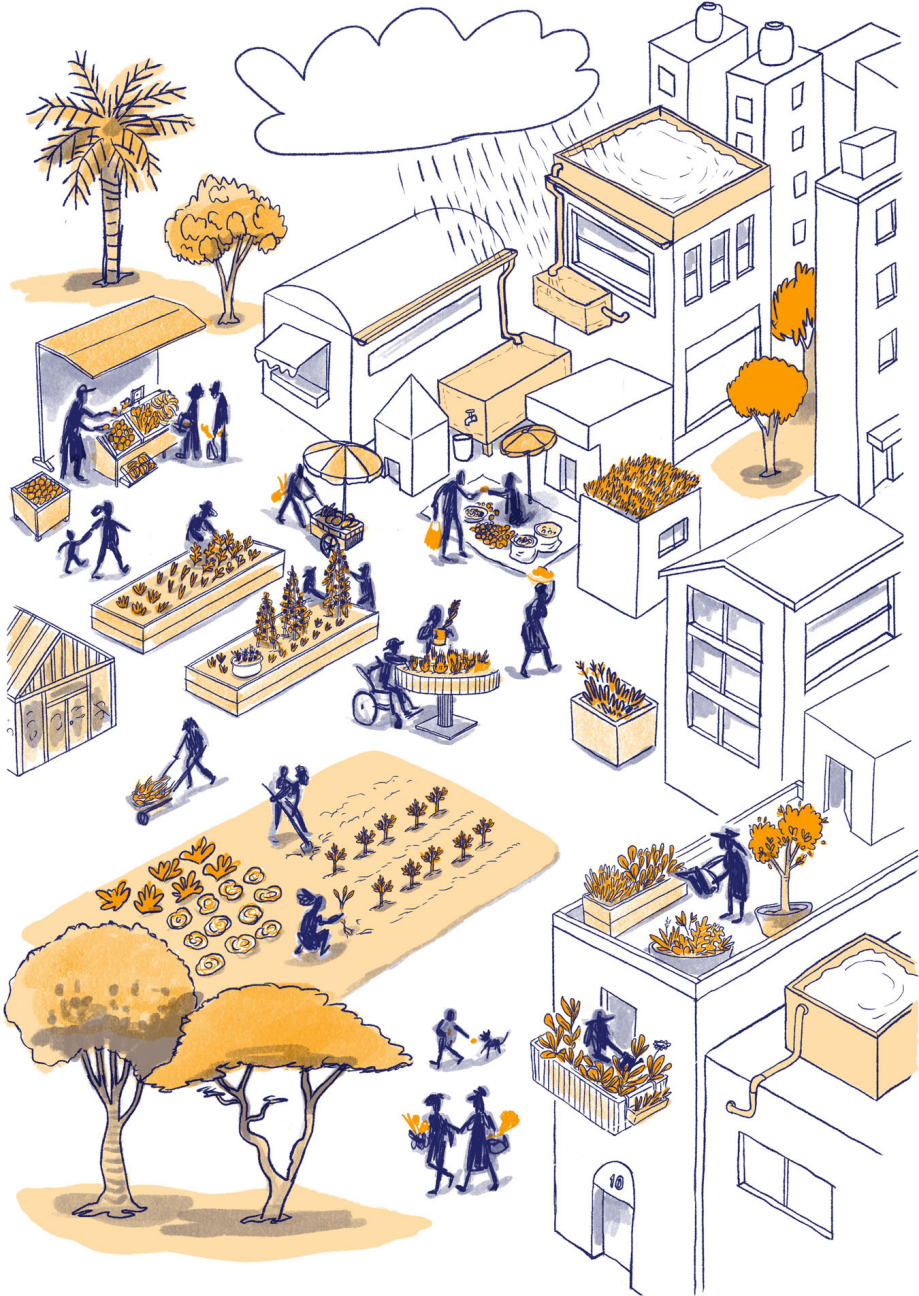
The final illustration representing urban farming. This illustration portrays diversity in architecture, vegetation, and transportation and selling of goods. In our project, the inclusion of different street vendors (including fixed and temporary, formal and informal, such as market stalls or street hawkers) achieved the necessary geographical and cultural diversity. Furthermore, the pre-pilot sketch of this illustration depicted people with full features, rather than stick people. We observed, however, that the full features generated an important debate about cultural representation that was not intended on our side. Substituting this with stick figures moved the focus away from the people and onto the practice of urban farming and the importance of green infrastructure. © All rights reserved. Illustration: Josune Urrutia.

An image does not have a unique language, but it needs to be ‘read’ or interpreted by multiple audiences with different cultural backgrounds. This art–science collaboration aimed to design visuals of adapting cities that could be anywhere in the world and yet familiar to all participants. A risk is to create artificial realities that are no longer realities for anybody involved. Reviewing and redesigning the images involved a great deal of research about different architectures, people, vegetation, and most significantly, different forms of street life and street activities (see Fig. 1 and the representation of different street vendors).

### Symbolic elements

Visuals are created based on symbolism because this helps the user connect more easily to their own experiences. Sometimes, these very symbols might be interpreted equally but have different meanings across contexts, producing different reactions regarding how good or bad adaptation might be. For example, green infrastructure can be seen as a utopian transformative solution or as a source of social and economic concerns (allergies, sanitation, water use, economic costs, gentrification). While the diversity of interpretations can generate rich dialogues, the challenge more severely emerges when adaptation processes and practices are illustrated (justice, institutionalisation, uncertainty). In this case, we found the choice of symbols critical because of the strong social, cultural, and political weight of context-specific adaptation practices.

### Simplicity and detail

We have also encountered trade-offs between simplicity and detail. While simplicity might remove the complexity needed to evoke experiences, too much detail can also generate distraction and misinterpretation (see Fig. 1 and the representation of people). However, distraction and misinterpretation should not be so easily discarded since they might indicate different epistemological frameworks. We should then ask ourselves why those images were not interpreted as expected and what the implications are for our local understanding of adaptation.

### Scientific method and piloting

**Fig. 2:**
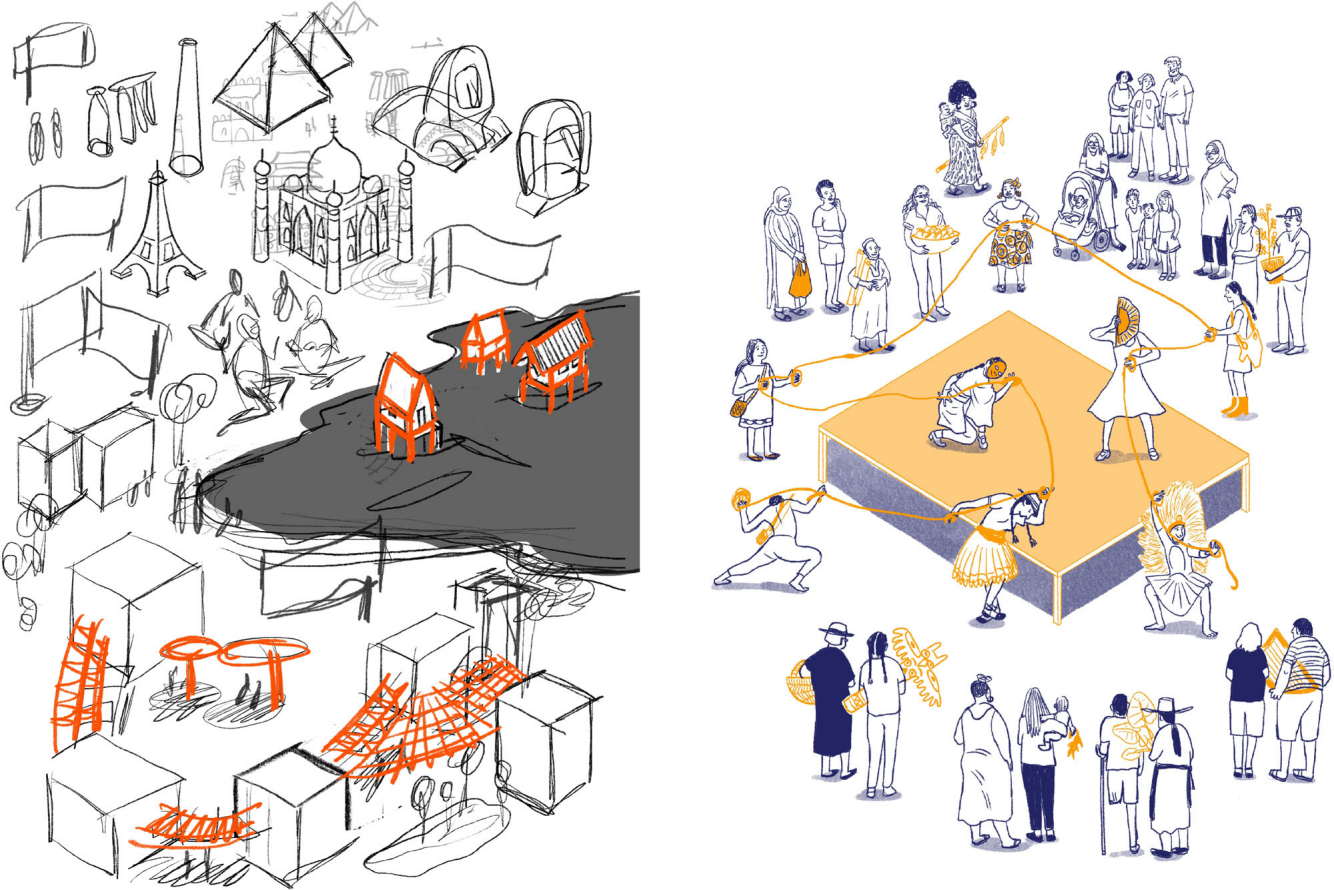
An initial sketch (to the left) of the final illustration (to the right) representing cultural heritage in relation to climate adaptation. The initial sketch focused on iconographical examples of cultural heritage. Following the development of the initial sketch, the team realised that whilst cultural heritage is often visualised through physical infrastructure, the importance of cultural heritage is the role it can play in the resilience and cohesion of local communities. By working with the artist, we shifted the focus to the intangible aspect or the thread that forms the foundation of local communities’ identity and can make them more resilient to climate change. © All rights reserved. Illustrations: Josune Urrutia.

Our process followed the scientific method and included a pilot of the illustrations. Working with the artist to visualise scientific understandings and take on the feedback from the pilot participants required a space of respect and trust. The process was complicated and required several rounds of comments and feedback to achieve images that resonated with our very diverse target audience, and that represented the ‘right’ aspect of adaptation (see Fig. 2). This arduous, but necessary process represented a battle of knowledge. Balancing the views of pilot participants with the artist’s expertise and experience can easily become a trap that reproduces the very knowledge dominance that our work tries to break down.

All in all, ensuring that there are adequate resources for the whole creative and scientific process was a lesson that we learnt: time and suitable space for discussion and research were critical to the six-month process of concept identification and the creation of the illustrations.

New mechanisms for adaptation management, such as art-based approaches, can capture the complexity of experiences, identify diverse understandings, create common ones and open the door for different perspectives that are inherently necessary to deglobalise adaptation narratives and establish goals that count across individuals and contexts. From our experience, future research on the means and outcomes of art–science collaborations should place greater emphasis not only on how the arts can be used for scientific dissemination but, more importantly, on how they can be used for scientific inquiry and the elicitation of multiple knowledges. Given the current universalisation of the concept, adaptation to climate change makes the perfect case.

## References

[1] IPCC. In *Climate Change 2022: Impacts, Adaptation and Vulnerability. Contribution of Working Group II to the Sixth Assessment Report of the Intergovernmental Panel on Climate Change* [eds Pörtner, H.-O. et al.) 2897–2930 (Cambridge University Press, 2022).

[2] Eriksen S (2021). Adaptation interventions and their effect on vulnerability in developing countries: help, hindrance or irrelevance?. World Dev..

[3] Schipper ELF (2020). Maladaptation: when adaptation to climate change goes very wrong. One Earth.

[4] Juhola S, Glaas E, Linnér B-O, Neset T-S (2016). Redefining maladaptation. Environ. Sci. Policy.

[5] Dilling L (2019). Is adaptation success a flawed concept?. Nat. Clim. Change.

[6] Rahman, M. F. et al. Locally led adaptation: promise, pitfalls, and possibilities. *Ambio* **52**, 1543–1557 (2023). 10.1007/s13280-023-01884-7 PMC10460758 37286919

[7] Vincent K (2023). Development geography II: Community-based adaptation and locally-led adaptation. Prog. Hum. Geogr..

[8] Goonesekera SM, Olazabal M (2022). Climate adaptation indicators and metrics: state of local policy practice. Ecol. Indic..

[9] Olazabal M, Ruiz De Gopegui M (2021). Adaptation planning in large cities is unlikely to be effective. Landsc. Urban Plan..

[10] Reckien D (2023). Quality of urban climate adaptation plans over time. npj Urban Sustain..

[11] Anguelovski I (2016). Equity impacts of urban land use planning for climate adaptation: critical perspectives from the global north and south. J. Plan. Educ. Res..

[12] Singh C (2022). Interrogating ‘effectiveness’ in climate change adaptation: 11 guiding principles for adaptation research and practice. Clim. Dev..

[13] Biesbroek R, Lesnikowski A (2023). Unpacking symbolic policy-making for the first Global Stocktake under the Paris Agreement. npj Clim. Action.

[14] Clark SE (2020). 6&6: a transdisciplinary approach to art–science collaboration. Bioscience.

[15] O’Neill S (2020). More than meets the eye: a longitudinal analysis of climate change imagery in the print media. Clim. Change.

[16] O’Neill SJ, Boykoff M, Niemeyer S, Day SA (2013). On the use of imagery for climate change engagement. Glob. Environ. Change.

[17] Collaborations with artists go beyond communicating the science. *Nature* **590**, 528–528 (2021). 10.1038/d41586-021-00469-2 33627816

